# Functional elucidation of the non-coding RNAs of *Kluyveromyces marxianus* in the exponential growth phase

**DOI:** 10.1186/s12864-016-2474-z

**Published:** 2016-02-29

**Authors:** Yoo-Bok Cho, Eun Ju Lee, Suhyung Cho, Tae Yong Kim, Jin Hwan Park, Byung-Kwan Cho

**Affiliations:** Department of Biological Sciences and KI for the BioCentury, Korea Advanced Institute of Science and Technology, Daejeon, 305-701 Republic of Korea; Biomaterials Lab., Samsung Advanced Institute of Technology (SAIT), 130 Samsung-ro, Yeongtong-gu, Suwon 443-803 Republic of Korea; Intelligent Synthetic Biology Center, Daejeon, 305-701 Republic of Korea

**Keywords:** *Kluyveromyces marxianus*, Non-coding RNA, Long antisense ncRNA, RNA-seq, Pervasive transcription, Metabolism

## Abstract

**Background:**

Non-coding RNAs (ncRNAs), which perform diverse regulatory roles, have been found in organisms from all superkingdoms of life. However, there have been limited numbers of studies on the functions of ncRNAs, especially in nonmodel organisms such as *Kluyveromyces marxianus* that is widely used in the field of industrial biotechnology.

**Results:**

In this study, we measured changes in transcriptome at three time points during the exponential growth phase of *K. marxianus* by using strand-specific RNA-seq. We found that approximately 60 % of the transcriptome consists of ncRNAs transcribed from antisense and intergenic regions of the genome that were transcribed at lower levels than mRNA. In the transcriptome, a substantial number of long antisense ncRNAs (lancRNAs) are differentially expressed and enriched in carbohydrate and energy metabolism pathways. Furthermore, this enrichment is evolutionarily conserved, at least in yeast. Particularly, the mode of regulation of mRNA/lancRNA pairs is associated with mRNA transcription levels; the correlation between the pairs is positive at high mRNA transcriptional levels and negative at low levels. In addition, significant induction of mRNA and coverage of more than half of the mRNA sequence by a lancRNA strengthens the positive correlation between mRNA/lancRNA pairs.

**Conclusions:**

Transcriptome sequencing of *K. marxianus* in the exponential growth phase reveals pervasive transcription of ncRNAs with evolutionarily conserved functions. Studies of the mode of regulation of mRNA/lancRNA pairs suggest that induction of lancRNA may be associated with switch-like behavior of mRNA/lancRNA pairs and efficient regulation of the carbohydrate and energy metabolism pathways in the exponential growth phase of *K. marxianus* being used in industrial applications.

**Electronic supplementary material:**

The online version of this article (doi:10.1186/s12864-016-2474-z) contains supplementary material, which is available to authorized users.

## Background

The haploid and thermotolerant *Kluyveromyces marxianus* is a non-conventional yeast species with several advantageous metabolic properties over *Saccharomyces cerevisiae,* such as fermentation ability at high temperatures, ability to grow on various hexose and pentose sugars, production of less ethanol in the presence of excessive sugar, and weak glucose repression, which enables the fermentation of mixed sugars, such as hemicellulose hydrolysate and inulin, at higher temperatures [1]. These properties facilitate the development of efficient fermentation processes utilizing *K. marxianus*. As a result, this Generally Regarded As Safe (GRAS) species shows potential for use as a cell factory with high capability for improving biomass yields in industrially relevant biotechnological applications. For example, *K. marxianus* has been utilized for the reduction of lactose content in food products as well as for the production of ethanol, various enzymes, heterologous proteins, aromatic compounds, and bioingredients, and for bioremediation [2].

In order to engineer this species to be more suitable for use in various applications, genetic resources, such as its genome and transcriptome, are required at the genomic scale. *K. marxianus* is a member of the Saccharomycetales; however, the genetics and metabolism of this yeast are considered quite different from those of *S. cerevisiae* from an evolutionary point of view [1]. For instance, the mode of regulation of genes in the glycolysis and tricarboxylic acid (TCA) cycle pathways differs between the two yeast species, although they are largely conserved [3]. In particular, regulatory mechanisms in metabolic networks governing carbon assimilation have not yet been explored. It has been established that transcriptional regulatory networks, comprising of transcription factors and other auxiliary components, control metabolic flexibility and robustness in response to environmental conditions. Therefore, a full understanding of the cellular response to growth conditions as well as the roles of cognate regulators, such as transcription factors, is necessary for the elucidation of changes in transcript levels of metabolic genes due to the effects of growth conditions.

Interestingly, genome-wide transcriptome analyses have demonstrated that the eukaryotic genome is pervasively transcribed [4], e.g., more than 85 % of the genome of *S. cerevisiae* is transcribed [5]. This is due to a plethora of previously unannotated non-coding RNAs (ncRNAs), which are pervasively transcribed from intergenic and antisense regions of annotated genes. As transcription requires a large amount of cellular energy, nonfunctional pervasive transcription may impose a metabolic and regulatory burden on cells. In accordance with this, diverse functions of ncRNAs, such as in the modulation of gene expression related with metabolism and pathogenesis, have been revealed [4, 6]. However, there have been few reports on the functional characterization of ncRNAs in non-model yeast, despite accumulating evidence of the roles of regulatory ncRNAs in model organisms [6–8]. The evolutionary conservation of antisense RNA (asRNA) has been reported between *S. cerevisiae* and *Saccharomyces paradoxus,* which are in the *sensu stricto* Saccharomycetales [9, 10]. Additionally, the evolutionary conservation of long asRNAs between five *sensu stricto* Saccharomycetales members and *Kluyveromyces lactis* has been reported [11]. Thus, the evolutionary conservation of pervasive ncRNA transcription in budding yeasts suggests that these ncRNAs perform important functions in these organisms [9, 10].

In order to examine the functions and extent of transcription of ncRNA, we analyzed the transcriptomic changes at three time points during the exponential growth phase in *K. marxianus* by conducting strand-specific RNA-seq. The results indicated pervasive ncRNA transcription in the exponential growth phase. Additionally, we performed enrichment analysis of differentially expressed transcripts to demonstrate that long antisense ncRNAs (lancRNAs) show functional associations with carbohydrate and energy metabolism. The correlation between transcription levels in mRNA and lancRNA pairs suggests potential mechanisms by which these RNAs perform their functions.

## Results

### RNA-seq at the exponential growth phase

**Fig. 1:**
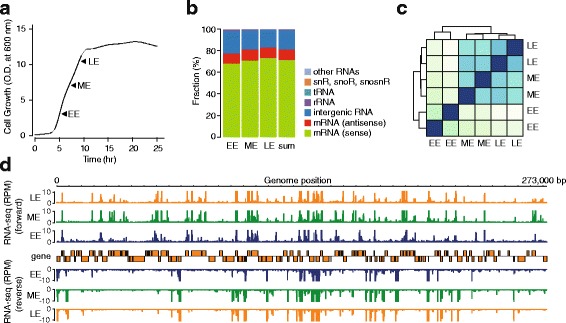
Genome-wide measurement of transcriptome during exponential growth phase. **a**–**e** EE, ME, and LE indicate early-exponential (EE), mid-exponential (ME), and late-exponential (LE) growth phase, respectively. **a** *K. marxianus* growth curve in YNB-u medium. RNA collection points at exponential growth phase (EE, ME, and LE) are indicated by *arrows*. **b** Reads mapping fraction of the three experimental conditions against location relative to gene classes. **c** Heatmap of hierarchical clustering among the RNA-seq experiments of the two biological replicates of the three experimental conditions, which was carried out by DESeq with variance-stabilizing transformation function. **d** RNA-seq profile of example genomic region during cell growth. Data for each condition were normalized to RPM (reads per million reads) to make y-axes same scale

We are particularly interested in transcriptional regulation at exponential growth phase where most biomass production is accomplished. In order to measure dynamic transcriptomic changes during exponential growth in the non-model yeast *K. marxianus* and achieve further understanding of the cellular response to the exponential growth conditions, we sequenced total RNAs isolated at three time points corresponding to early-exponential (EE), mid-exponential (ME), and late-exponential (LE) growth phase, with two biological replicates for each sample (Fig. 1a). We employed the dUTP method for RNA-seq [12] and obtained 23,309,796 mapped reads for EL, 28,076,013 mapped reads for ML, and 37,484,281 mapped reads for LL (Additional file 1: Table S1) [13]. These corresponded to 68.6 % of the genome being transcribed from one strand of DNA and 17.2 % being transcribed from both strands of DNA. Considering only the gene region, 68.8 % of the sense strand and 30.5 % of the antisense strand were transcribed, and 24.8 % of the gene regions were transcribed from both strands. Most of the sequence reads were mapped to the sense strand of protein coding genes (~70 %), intergenic regions (~20 %), and antisense strand of protein coding genes (~10 %) (Fig. 1b). A low number of sequence reads (<1.2 %) were mapped to rRNAs, indicating that rRNA depletion was successfully carried out. During cell growth, the fraction of reads mapped to the sense strand of protein coding genes was increased (68.2 % → 71.0 % → 73.4 %), while that mapped to the intergenic region was decreased (20.5 % → 17.7 % → 16.3 %). The fraction of reads mapped to the antisense strand of protein coding genes was almost unchanged (9.5 % → 10.3 % → 9.6 %). This result suggested that not only mRNA, but also a substantial amount of ncRNAs, were changed to achieve rapid cell growth in the exponential phase. Hierarchical clustering of biological replicates showed that, overall, experimental procedures were reproducibly conducted (Fig. 1c). In particular, the transcriptional landscape of RNA-seq indicated high strand-specificity and distinct transcriptional expression patterns during cell growth (Fig. 1d).

### Pervasive ncRNA transcription across the genome

**Fig. 2:**
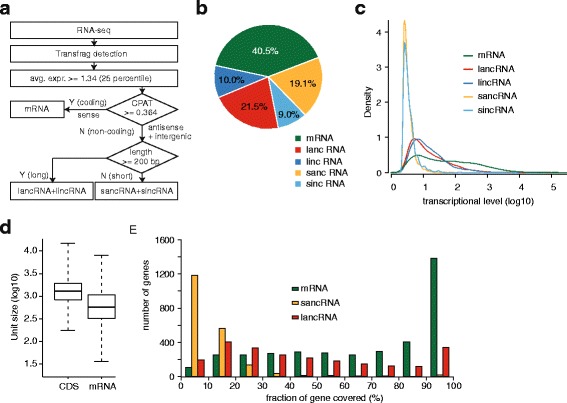
Detection and classification of transcriptional unit (transfrag). **a** Schematic demonstration of analytical procedures for transfrag detection and classification. CPAT program was used for predicting coding potential. **b** Proportion of transfrag classes among the detected transfrags. **c** Distribution of transcription level of transfrag classes. Transcriptional level was obtained from DESeq result. **d** Length distribution of protein-coding genes (CDS) and mRNA. **e** Distribution of fraction of gene model covered by mRNA, lancRNA, and sancRNA

We annotated 4839 protein coding genes from the *K. marxianus* genome using AUGUSTUS (Additional file 2: Table S2) [13, 14]. Gene units were defined without taking exon-intron structures into account, as the number of introns in the *K. marxianus* genome is less than 5 % [15]. Cmsearch program in Infernal version 1.1 [16] yielded 273 RNA genes using Rfam data (Additional file 3: Table S3) [17]. Subsequently, we obtained transcription units across the genome by transfrag method and subsequent post-processing (Fig. 2a). Transfrag is defined as contiguous genomic region actively transcribed [18]. Briefly, we discarded transfrags of transcriptional level lower than 1.34 (25 percentile) to reduce false positives in the detected transcripts. In addition, transfrags overlapping with either the forward strand or reverse strand of RNA genes were removed in order to focus on the ncRNAs associated with protein-coding genes. We then classified the transfrags into five RNA classes based on their length (short: length <200 nt and long: length ≥200 nt) [7], coding potential (non-coding: CPAT coding potential <0.364 and coding: CPAT coding potential ≥0.364) (Additional file 4: Figure S1) [19], and location relative to gene annotation (sense, antisense, and intergenic) [7]: (1) mRNA (sense transfrag with coding potential) (2) long antisense ncRNA (lancRNA), (3) long intergenic ncRNA (lincRNA), (4) short antisense ncRNA (sancRNA), and (5) short intergenic ncRNA (sincRNA). We obtained 14,298 transfrags, corresponding to 5785 mRNAs (40.5 %; average length = ~802 bp), 3067 lancRNAs (21.5 %; average length = ~538 bp), 1430 lincRNAs (10.0 %; average length = ~456 bp), 2726 sancRNAs (19.1 %; average length = ~117 bp), and 1290 sincRNAs (9.0 %; average length = ~117 bp) (Fig. 2b; Additional file 5: Table S4). These data demonstrate pervasive ncRNA transcription, which comprised ~60 % of transfrags with ~30 % of mapped reads (Fig. 1b).

Most ncRNA had a lower transcriptional level than mRNAs, although both lancRNA and lincRNA had higher transcriptional level than both sancRNA and sincRNA (Fig. 2c) [9, 10, 20]. The average length of mRNA transfrags was ~802 bp, which is about half the length of protein-coding genes (average length = ~1545 bp) (Fig. 2d). Among the protein-coding genes with mRNA transfrags (78.3 %; 3788 genes), 66.7 % of genes had only one transfrag and 88.2 % of genes had no more than two transfrags. In addition, the number of genes covered by transfrags indicated that the majority of mRNA transfrags covered more than 90 % of the gene annotation (Fig. 2e). Although lancRNA and sancRNA transfrags covered much fewer genes than mRNA transfrags, a substantial proportion of the gene region was covered by lancRNAs. Approximately 40 % of lancRNAs covered more than 50 % of the gene annotation while only ~2.5 % of sancRNA covered the same proportion. Taken together, no more than two transfrags were detected for most genes, and thus over-fragmentation of transcripts into multiple transfrags was negligible.

In principle, the 5′- and 3′-end position of each transfrag represents the transcription start site (TSS) and transcription termination site (TTS), respectively. These genomic features enabled us to determine whether the transfrags contained artifacts. In order to test this, we compared mapped read enrichment for each RNA class of *K. marxianus* with those of *S. cerevisiae*, sampled at exponential phase [9]. The comparison showed that the mapped read enrichment of all RNA classes was highly similar to that of *S. cerevisiae*, suggesting that the transfrags are highly accurate and ncRNAs are pervasively transcribed in *K. marxianus* (Additional file 6: Figure S2). Unexpectedly, lincRNAs and sincRNAs also showed high levels of transcription at the opposite strand, with a much lower transcriptional level than those of lancRNAs and sancRNAs. The proportion of intergenic ncRNA region covered by antisense transcription was ~26.0 %. In accordance with this, several cases of antisense transcription in ncRNAs have been reported [21, 22].

### Regulatory roles of ncRNA

**Fig. 3:**
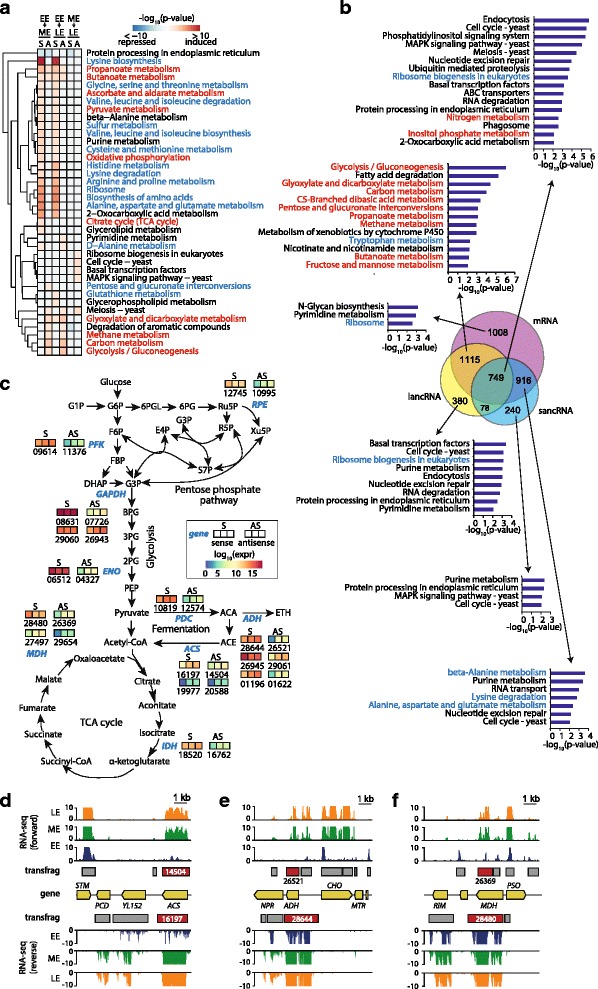
Enrichment of KEGG pathways. **a** Overlap of genes with mRNA, lancRNA, and sancRNA and their enriched KEGG pathways. *Blue colored letters* indicate amino acid metabolism pathways and *red colored letters* indicate carbohydrate metabolism or energy metabolism pathways. **b** Heatmap of significantly enriched KEGG pathways by differentially expressed sense and/or antisense transfrags. Amino acid metabolism pathways are indicated in blue lettering and carbohydrate metabolism or energy metabolism pathways are indicated in red. Three consecutive rectangular demonstrate transcriptional level of EE, ME, and LE conditions of sense and antisense transcription, respectively. **c** Genes with differentially expressed lancRNAs at core carbohydrate metabolic pathway. **d** RNA-seq profile near ACS. **e** RNA-seq profile near ADH. **f** RNA-seq profile near MDH

In order to investigate whether pervasive ncRNA transcription at the exponential growth phase plays a functions, we focused on mRNA, lancRNA, and sancRNA, as the functions of genes within these RNA classes may be simply inferred from gene annotation [20]. By using DESeq for EE to ME condition and EE to LE condition (*p*-value < = 0.05), we obtained 3572 differentially expressed transfrags, comprising of 2449 sense, 615 antisense, and 508 intergenic transfrags (Additional file 7: Figure S3) [23]. From these transfrag pairs, significantly enriched KEGG pathways were separately obtained for sense and antisense strands (Fig. 3a) [10, 24]. These results demonstrated enrichment of carbohydrate metabolism, including glycolysis and amino acid biosynthesis pathways as well as respiration pathways. These pathways are important for the synthesis of fundamental cellular components and energy production to fulfill energy requirements for rapid growth during the exponential phase [25, 26]. However, we observed gradual inactivation of respiration-related pathways, such as the TCA cycle and oxidative phosphorylation during cell growth. The gradual inactivation of the pathways indicates that aerobic condition had been changed to anaerobic condition due to a decrease in dissolved oxygen levels [27]. This pathway enrichment pattern at the exponential phase is consistent with the fact that *K. marxianus,* being a Crabtree-negative species, uses aerobic-respiration, thereby producing energy from carbon sources [25]. Interestingly, the enriched pathways could be categorized into three groups based on the differential expression of sense, antisense, or both strands. Each group showed distinct functional associations; genes with differential expression of only the sense strand were mostly associated with amino acid metabolism and energy production related to mitochondrial respiration, whereas those with differential expression of antisense strands only, or both strands, were associated with mostly carbohydrate metabolism. The asRNA-mediated regulation of carbohydrate metabolism was mostly conducted by lancRNA (Fig. 3b).

In particular, several differentially expressed lancRNAs were located in core carbohydrate metabolic genes, many of which were found to be important for the regulation of their constituent pathways (Fig. 3c). There were three genes in the glycolysis pathway, *PFK*, *GAPDH*, and *ENO,* for which antisense transcription was significantly induced. Antisense transcription of the *PFK* gene was significantly induced under ME and LE conditions; however, transcription from the opposite sense strand was not induced under these conditions. Considering *PFK* gene encodes a rate-limiting enzyme of glycolysis in yeast and human cancer cells, it is highly regulated at the transcriptional level by ncRNA [28, 29]. We detected two *GAPDH* homologs that showed significant induction of antisense transcription at ME and LE conditions; however, no transcription induction was observed in their sense strands. The *GAPDH* gene encodes a key glycolytic enzyme and functions as a metabolic switch to reroute carbohydrate flux to protect against oxidative stress [30]. The *ENO* gene encodes one of the most highly expressed glycolytic enzymes in many organisms [31] whose activity is known to be regulated by gene expression to a very low extent. We found that the transcription from the sense strand of the *ENO* gene was slightly reduced, while that from the antisense strand was significantly induced. The antisense strands of two genes, *IDH* and *MDH,* which encode enzymes of the TCA cycle, were significantly induced. The *IDH* gene encodes a rate-limiting enzyme of the TCA cycle. Antisense transcription of the *IDH* gene was significantly induced while sense transcription was slightly increased. MDH catalyzes the final step of the TCA cycle (conversion of malate into oxaloacetate) [31, 32]. Antisense strands were significantly induced in two *MDH* homologs; however, antisense transcription was increased in one homolog but decreased in the other. This suggests that each homolog is under distinct antisense-mediated transcriptional regulation.

We observed that three fermentation genes, *PDC*, *ADH*, and *ACS*, showed significant induction of antisense strands. Induction of the fermentation genes is consistent with the inactivation of the TCA cycle and oxidative phosphorylation genes in Crabtree-negative species [27]. *PDC* encodes a key enzyme of alcoholic fermentation, which cleaves pyruvate into carbon dioxide and acetaldehyde, and is auto-regulated [33]. Antisense transcription of the *PDC* gene was significantly induced and sense transcription was concordantly increased with antisense transcription. The antisense strands of three *ADH* homologs, which are responsible for conversion alcohol into aldehyde in *S. cerevisiae*, were significantly induced. Two of the sense strands were significantly induced and one was significantly repressed. The ACS enzyme is responsible for the transformation of acetate into acetyl-CoA. We detected differentially expressed transfrags from two *ACS* homologs. In one homolog, both strands were significantly induced whereas in the other, only the sense strand was significantly induced. The RPE enzyme, a constituent of the pentose phosphate pathway, is responsible for the conversion of ribulose 5-phosphate into xylulose 5-phosphate. Both the sense and antisense strands of the *RPE* gene were differentially expressed.

Among the genes described above, we selected three genes, *ACS*, *ADH*, and *MDH*, for the investigation of their antisense transcription pattern. RNA-seq profiles of the genes demonstrated an increase in transcription from both sense and antisense strands under ME and LE conditions, which indicated concordant increase of transcriptional level of mRNA/lancRNA pairs (Fig. 3d–f). Furthermore, they showed obvious strand specificity, except in genes with lancRNA. These demonstrate that their antisense transcription is transcribed and their transcriptional level is simultaneously increased indeed.

### Mode of regulation of mRNA/lancRNA pairs

A given lancRNA can exert both positive and negative regulation of its cognate mRNA [34, 35]. Accordingly, we had observed both cases. In order to investigate mode of regulation, we compared the transcriptional levels of mRNA/lancRNA pairs, where either one or both of the pairs were differentially expressed under EE, ME, and LE conditions. LancRNAs regulate their target by base-pairing [20] and this suggests that interaction between mRNA and lancRNA may be associated with the mode of regulation. Therefore, we hypothesized that the following two factors are associated with mode of regulation, (1) three differential expression types of mRNA/lancRNA pairs, as follows: differentially expressed mRNA/differentially expressed lancRNA pairs, differentially expressed mRNA/non-differentially expressed lancRNA pairs, and non-differentially expressed mRNA/differentially expressed lancRNA pairs, and (2) length fraction of mRNA covered by lancRNA.

**Fig. 4:**
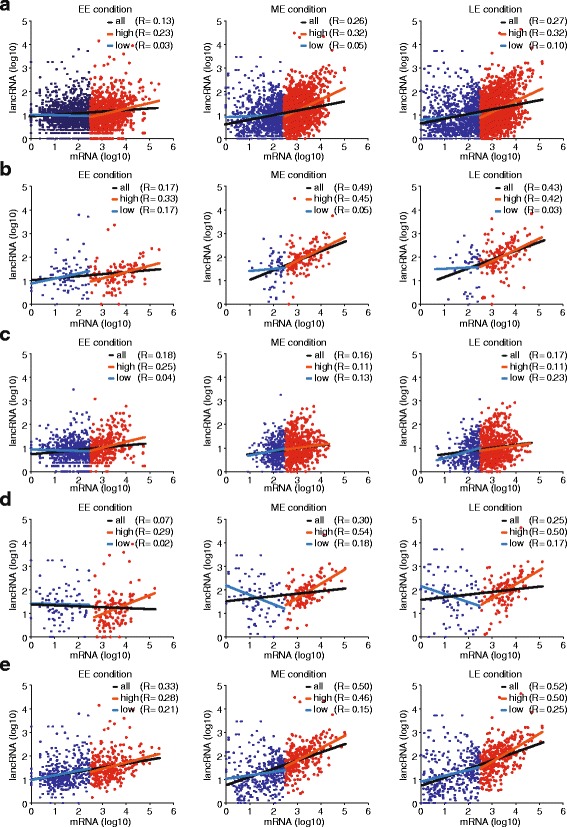
Correlation between transcriptional level of mRNA and lancRNA pairs. **a** All mRNA/lancRNA pairs where either one or both member of each pair was differentially expressed during EE, ME, and LE. **b** Differentially expressed mRNA/differentially expressed lancRNA pairs. **c** Differentially expressed mRNA/non-differentially expressed lancRNA pairs. **d** Non-differentially expressed mRNA/differentially expressed lancRNA pairs. **e** Pairs with more than 50 % of mRNA covered by a lancRNA. *Red points* represent genes with high transcription level while *blue points* represent genes with low transcription level. *Black*, *red*, and *blue lines* indicate least squares fitting of mRNA and lancRNA pairs

In order to test the former hypothesis, we compared transcriptional levels of sense and antisense strands for each differential expression type separately, and found that they showed distinct correlation patterns. Taking all the mRNA/lancRNA pairs into account, our results showed weak positive correlation between lancRNA and mRNA which was consistent with findings in *S. cerevisiae* (Fig. 4a) [10]. Differentially expressed mRNA/differentially expressed lancRNA pairs demonstrated strong positive correlation whereas differentially expressed mRNA/non-differentially expressed lancRNA demonstrated weak positive correlation (Fig. 4b, c). The strong positive correlation indicates that transcriptional expression level of mRNA is co-regulated with lancRNA for each mRNA/lancRNA pairs. Interestingly, non-differentially expressed mRNA/differentially expressed lancRNA pairs showed unexpected results (Fig. 4d). Although a weak positive correlation was observed when all the pairs were taken into account, they showed obvious negative correlation at low mRNA transcription level while positive correlation at high mRNA transcription level. This shows that a certain threshold of transcriptional level of cognate mRNA is important to determine the mode of enhancing or repressing by lancRNAs. Thus, we concludes that this was the result of switch-like behavior of lancRNA, as negative regulation at low mRNA transcription levels could be interpreted as ensuring the “off” state of mRNA transcription, and vice versa [36]. Thus, our results suggest that the transcriptional mode of regulation of lancRNA was influenced by differential expression types of the pairs and mRNA transcriptional levels.

**Fig. 5:**
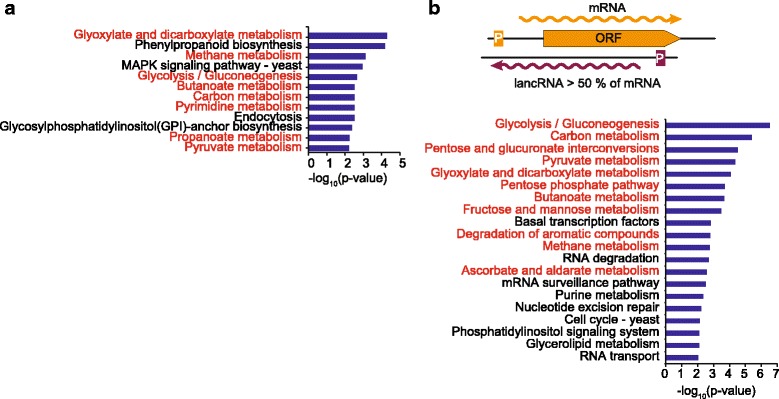
Significantly enriched KEGG pathways of genes with lancRNAs. **a** Significantly enriched KEGG pathways of genes with all lancRNAs. **b** Significantly enriched KEGG pathways of genes with lancRNA which covers more than half of coding region. *Red words* indicate carbohydrate metabolism or energy metabolism pathways

In order to test the latter hypothesis, we compared the transcriptional level of mRNA/lancRNA pairs in which more than 50 % of an mRNA was covered by a lancRNA (Fig. 4e). The result showed a positive correlation stronger than that observed when the covering length fraction was not considered (Fig. 4a). Furthermore, genes for which more than 50 % of the mRNA was covered by a lancRNA exhibited more obvious enrichment of carbohydrate or energy metabolic pathways (Fig. 5a, b). These data suggest that the covering length fraction is also an important factor in determining the transcriptional mode of regulation of lancRNA.

## Discussion

Pervasive ncRNA transcription, which has been demonstrated in the model organism *S. cerevisiae*, is evolutionarily conserved in the *sensu stricto* Saccharomycetales [9, 10]. Consistent with this, our results indicate that pervasive ncRNA transcription, from antisense and intergenic regions, also occurs in *K. marxianus* (Fig. 2b). It was found that ncRNAs accounted for ~60 % of all identified transfrags. Additionally, 77.5 % of protein-coding transfrags were found to possess either long or short ncRNAs at the opposite strand. Similar length fractions of genes (73.4 %) had ncRNAs at the opposite strand when only expressed genes were considered. In *S. cerevisiae*, there are large numbers of unannotated cryptic unstable transcripts (CUTs) and Xrn1-sensitive unstable transcripts (XUTs), which are destabilized after synthesis [37, 38]. Furthermore, CUTs are reported to be transcribed from both intergenic and antisense regions [37]. These data suggest that a large length fraction of ncRNAs in *K. marxianus* may be associated with CUTs and XUTs.

Our results show that lancRNA-mediated regulation is enriched for carbohydrate metabolism pathways (Fig. 3a, b). Enrichment analysis for lancRNAs covering more than half of the protein-coding genes in *S. cerevisiae* revealed a similar enrichment pattern of carbohydrate and energy metabolic pathways (e.g. carbon metabolism, glycolysis/gluconeogenesis, and pyruvate metabolism at mid-exponential phase) (Additional file 8: Figure S4) [10]. The evolutionary conservation of pathway enrichment suggested that pervasive ncRNA transcription plays evolutionarily conserved functions in *K. marxianus* [9, 10]. The importance of these pathways for rapid growth via the synthesis of fundamental cellular components and energy production [25, 26] suggests that lancRNAs may play major role in rapid growth during the exponential phase through currently unknown mechanisms.

Elucidation of the mode of regulation of lancRNA may provide insights into these unknown mechanisms. It is generally accepted that lancRNAs positively or negatively regulate their cognate mRNAs [34, 35]. A recent report proposed that lancRNAs function as on/off switches, thereby increasing the variability of gene expression [36]. Several cases of lancRNAs with on/off switch-like behavior have been reported [39–42]. Our results showed that mRNA/lancRNA pairs demonstrate inverse mode of regulation according to the transcriptional level of mRNA, especially in differentially expressed lancRNA/non-differentially expressed mRNA pairs (Fig. 4d), although the same trend was also observed in other types of mRNA/lancRNA pairs (Fig. 4b, c). Therefore, the relationship between transcriptional levels of mRNA and lancRNA involves switching mRNA transcriptional level between on and off states. In other words, lancRNAs enhance the transcriptional level of their cognate mRNAs if the mRNA transcriptional level is higher than certain threshold, but repress this if it is lower. Therefore, our results not only support the view of lancRNA functioning as an on/off switch, but also suggest that this represents a widely used mode of regulation, particularly in carbohydrate and energy metabolism pathways. In addition, our results showed that strong positive correlation exists between transcriptional levels of mRNA/lancRNA pairs, if both pairs are differentially expressed or if the length fraction of mRNA covered by lancRNA is more than 50 % (Fig. 4e). These data suggest that the two factors are important for enhancing mRNA transcriptional levels by a currently unknown mechanism. Consistent with this finding, a recent study showed that lancRNAs indeed play a role in enhancing mRNA transcriptional levels in several cases [43]. Taken together, our results suggest that a single lancRNA may play either a switch-like roles or an enhancing role depending on conditions such as the mRNA transcriptional level and differential expression of mRNA. However, the molecular mechanisms underlying this mode of regulation should be investigated in a specific candidate gene, as our findings are based on observations of the mRNA/lancRNA population. Besides, evolutionarily conserved enrichment of lancRNA differential expression, and several cases of lancRNA functioning as a switch for regulating mRNA in carbohydrate and energy metabolism pathways suggest that the switch-like function of lancRNA may be prevalent across a wide range of species.

Among genes with differentially expressed lancRNA, *PFK* encodes an enzyme that catalyzes fructose 6-phosphate (F6P) into fructose-1,6-bisphosphate (FBP) with the release of energy via ATP hydrolysis. *PFK* is one of the primary targets of glycolytic flux regulation according to ATP demand, and this regulation is conserved from bacteria to humans [44]. Reporter metabolites, such as ATP for *PFK*, play an important role in monitoring the environment or nutrient status by modulating the transcriptional level of associated genes [45–47]. Most genes with differentially expressed lancRNA are associated with cofactors used as reporter metabolites. The ACS enzyme also uses ATP whereas GAPDH, MDH, and PDC use NAD, and IDH uses NADP as cofactor. Recent studies show that long ncRNAs promote transcriptional poising of the immediate-early response of inducible genes [48, 49]. Therefore, the transcriptional status of these genes may serve as a good target for the regulation of glycolytic flux. Additionally, lancRNAs may enable rapid and efficient post-transcriptional switch in response to environmental changes, in contrast to metabolic regulation or gene regulation alone [44, 50]. Consistent with this, metabolic fluxes mediated by glycolytic enzymes are regulated at the post-transcriptional level [51, 52].

## Conclusion

In conclusion, *K. marxianus* transcribes ncRNAs pervasively during exponential growth. Among the ncRNA classes, lancRNAs are enriched for genes comprising carbohydrate or energy metabolism pathways. Further analysis of the correlation between mRNA and lancRNA suggests that lancRNAs enable switch-like behavior of their cognate mRNAs via transcriptional induction. Thus, lancRNA-mediated regulation of mRNA represents a mechanism for efficient regulation of carbohydrate and energy metabolism pathways.

## Methods

### Strains and culture conditions

*K. marxianus* var. marxianus ATCC 36907 (KM7) was obtained from the Korean Collection for Type Culture (KCTC) and grown in YBN-u media (0.67 % yeast nitrogen base without amino acids, uracil deprived amino acids, and 2 % (*w/v*) dextrose) at 30 °C in a shaking incubator [53]. Samples were taken at three time points corresponding to the EE (OD = ~ 3), ME (OD = ~ 7), and LE (OD = ~ 10) growth phases, with two biological replicates per sample.

### RNA isolation

Cells were harvested by centrifugation and resuspended in 300 μl lysis buffer (20 mM Tris‐HCl (pH 7.5), 140 mM NaCl, 5 mM MgCl_2_, and 1 % Triton‐X). Next, the resuspended cells were lysed with 1 mL TRIzol (Invitrogen) and incubated for 5 min at room temperature. After centrifugation for 15 min at 3000 rpm, the supernatant was transferred to a new tube and mixed with 200 μl of chloroform for 2–3 min. After another 15 min centrifugation step, the supernatant was mixed with triple volume of 100 % ethanol or equal volume of isopropanol, 2 μl of glycogen, and 3 M sodium acetate. After centrifugation and resuspension, RNA was washed with 70 % ethanol. After drying, RNA was resuspended in DEPC-treated water. In order to confirm the quality of extracted RNA, total RNA was visualized using agarose gel electrophoresis. The isolated RNA was incubated for 1 h at 37 °C with 4 U of rDNase I (Ambion) and 5 μl of 10× DNase I buffer (Ambion) for removal of genomic DNA. The DNA-free RNA was purified by phenol-chloroform extraction and ethanol precipitation.

### RNA-seq and data processing

Ribosomal RNA (rRNA) was removed by using Ribo-Zero Magnetic Gold Kit (Human/Mouse/Rat) (Epicentre) according to the manufacturer’s instructions. Two-hundred nanograms of mRNA was then fragmented by using 10× Fragmentation buffer (Ambion). The first strand cDNA was synthesized using the Random primers (Invitrogen) and SuperScript III Reverse Transcriptase (200 U/μl, Invitrogen). The second strand synthesis was done with *Escherichia coli* DNA polymerase (10 U/μl, Invitrogen), *E. coli* DNA ligase (10 U/μl, Invitrogen), and *E. coli* RNase H (2 U/μl, Invitrogen). The libraries for Illumina sequencing were constructed using TruSeq™ DNA Sample Prep Kit (Illumina) according to the manufacturer’s instructions. Briefly, the synthesized cDNA was end-repaired and 3′-ends of the blunt fragments were adenylated for adapter ligation. The adenylated DNA fragments were ligated with Illumina adapters. A fraction of the adapter-ligated DNA between 180 and 380 bp was size-selected from a 2 % agarose gel after electrophoresis. Size-selected DNA was purified by using MinElute Gel Extraction Kit (Qiagen) according to manufacturer’s instructions, and eluted in 1× TE buffer with low EDTA (10 mM Tris–HCl (pH 8.0), 0.1 mM EDTA) for the following enzyme reaction. For degradation of the second strand, which contains dUTP instead of dTTP, 1 U of USER enzyme (NEB) was added to the purified DNA and incubated for 15 min at 37 °C. After 5 min incubation at 95 °C for enzyme inactivation, the library was enriched by PCR. The amplification was monitored on a CFX96™ Real-Time PCR Detection System (Bio-Rad) and stopped at the beginning of the saturation point. The amplified library was purified by using Agencourt AMPure XP beads and quantified using a Qubit 2.0 fluorometer (Invitrogen). Finally, validated DNAs were sequenced using MiSeq (Illumina) and Miseq® V2 reagent kit of 50 cycles according to manufacturer’s manual. Sequenced reads were mapped to the genome sequence from NCBI (AKFM00000000.1) using CLC genomics workbench (masking mode = no masking, mismatch cost = 1, insertion cost = 3, deletion cost = 3, length fraction = 0.8, similarity fraction = 0.9, global alignment = yes, non-specific match handling = map randomly) after trimming of low quality regions [13]. RNA‐seq depth profiles were produced by in-house script and visualized using SignalMap (NimbleGen).

### Gene annotation

Due to the lack of gene annotation in *K. marxianus* genome, we predicted protein coding genes by using AUGUSTUS with default parameters and trained with gene set of *K. lactis* [54]. For RNA gene annotation, we predicted genes using cmsearch of Infernal (1.1rc4) and Rfam (version 12.0) [16, 17]. We discarded manually apparent non-yeast entries, such as bacteria, originating after discarding entries with *p*-value over 0.01. Gene name was obtained by BLASTP homology search to fungal proteome database.

### Transfrag identification and functional analysis

In order to obtain transcriptional units, we identified transfrags by combining RNA‐seq results for all three conditions. We merged nearby transfrags to reduce over-fragmentation if the distance between them was shorter than 40 bp. Nearby transfrags of distance range from 40 to 100 bp were merged if *p*‐value of Wilcoxon rank test with two‐sided was less than 10^−20^, assuming that they had statistically similar profiles. Finally, we discarded transfrags with transcriptional expression levels below 25 percentile transcriptional level from DESeq analysis to obtain *bona fide* ncRNAs [23]. Predicted transfrags were classified as sense, antisense, and intergenic, according to location, compared with annotated genes. If a transfrag covered more than two gene annotations, it was divided into multiple transfrags corresponding to sense, antisense, and intergenic transfrags. This process was conducted by in‐house script and manual inspection was followed. CPAT was used for calculation of coding potential of transfrags and for the discrimination of ncRNAs from protein-coding ones [19]. Cutoff value (0.364) for discrimination was determined by R script within the package as described, by using RNA genes as reference non-coding genes [19]. Differentially expressed transfrags were detected using DESeq [23]. For KEGG enrichment analysis, we linked KEGG pathway information by homology search to SwissProt, which has links to KEGG Orthology information, due to the lack of KEGG pathway annotation for *K. marxianus*. A pathway with a *p*‐value lower than 0.01, as determined by two‐tailed Fisher exact test, was considered enriched with statistical significance. To compare transcriptional level of mRNA/lancRNA pairs and mRNA/lincRNA pairs, we associated transfrag pairs if their genomic locations are overlapped. Correlation between transcriptional level of mRNA/lancRNA pairs and mRNA/lincRNA pairs were calculated by Pearson correlation coefficient.

### Availability of supporting data

The RNASeq dataset supporting the results of this article is available in the Gene Expression Omnibus (GEO) repository, [http://www.ncbi.nlm.nih.gov/geo/query/acc.cgi?acc=GSE70111].

## Additional files

Additional file 1: Table S1. Strand-specific RNA-seq. (XLSX 11 kb) Additional file 2: Table S2. Annotation of Kluyveromyces genome. (XLSX 286 kb) Additional file 3: Table S3. Identification of RNA genes using Rfam data. (XLSX 23 kb) Additional file 4: Figure S1. Determination of optimal cutoff value to differentiate non-coding transfrags from coding ones. The optimal cutoff value was determined by R script within the CPAT package with appropriate modification to fit our circumstance. (DOC 37 kb) Additional file 5: Table S4. Transfrag. (XLSX 664 kb) Additional file 6: Figure S2. Average read profile of each RNA classes. Solid line indicates forward strand while dotted line indicates reverse (antisense of the forward) strand. TIS indicates transcription initiation site and TTS indicates transcription termination site. (DOC 70 kb) Additional file 7: Figure S3. Differential expression of transfrags. (A) Differential expression pattern of mRNA/lancRNA pairs. (B) Differential expression pattern of mRNA/sancRNA pairs. (DOC 144 kb) Additional file 8: Figure S4. Significantly enriched KEGG pathways of genes with lancRNAs in *S. cerevisiae*. Significantly enriched KEGG pathways of genes with lancRNA which covers more than half of coding region at ME (mid exponential), ES (early stationary), and HS (heat shock) conditions. Red words indicate carbohydrate metabolism or energy metabolism pathways. In constrast to *K. marxianus* with no KEGG annotation, gene-pathway link information within KEGG annotation was used rather than inferred by homology search. (DOC 49 kb)

## Abbreviations

EE: early-exponential
ME: mid-exponential
LE: late-exponential
mRNA: messenger RNA
ncRNA: non-coding RNA
asRNA: antisense RNA
lancRNA: long antisense non-coding RNA
lincRNA: long intergenic non-coding RNA
sancRNA: short antisense non-coding RNA
sincRNA: short intergenic non-coding RNA
