# Supplementary material for: Functional elucidation of the non-coding RNAs of Kluyveromyces marxianus in the exponential growth phase
Source: BMC Genomics. 2016 Feb 29;17:154. doi: 10.1186/s12864-016-2474-z (PMC4770515; doi:10.1186/s12864-016-2474-z)
Supplement: Additional file 4: Figure S1. — Determination of optimal cutoff value to differentiate non-coding transfrags from coding ones. The optimal cutoff value was determined by R script within the CPAT package with appropriate modification to fit our circumstance. (DOC 37 kb) [file 12864_2016_2474_MOESM4_ESM.doc]

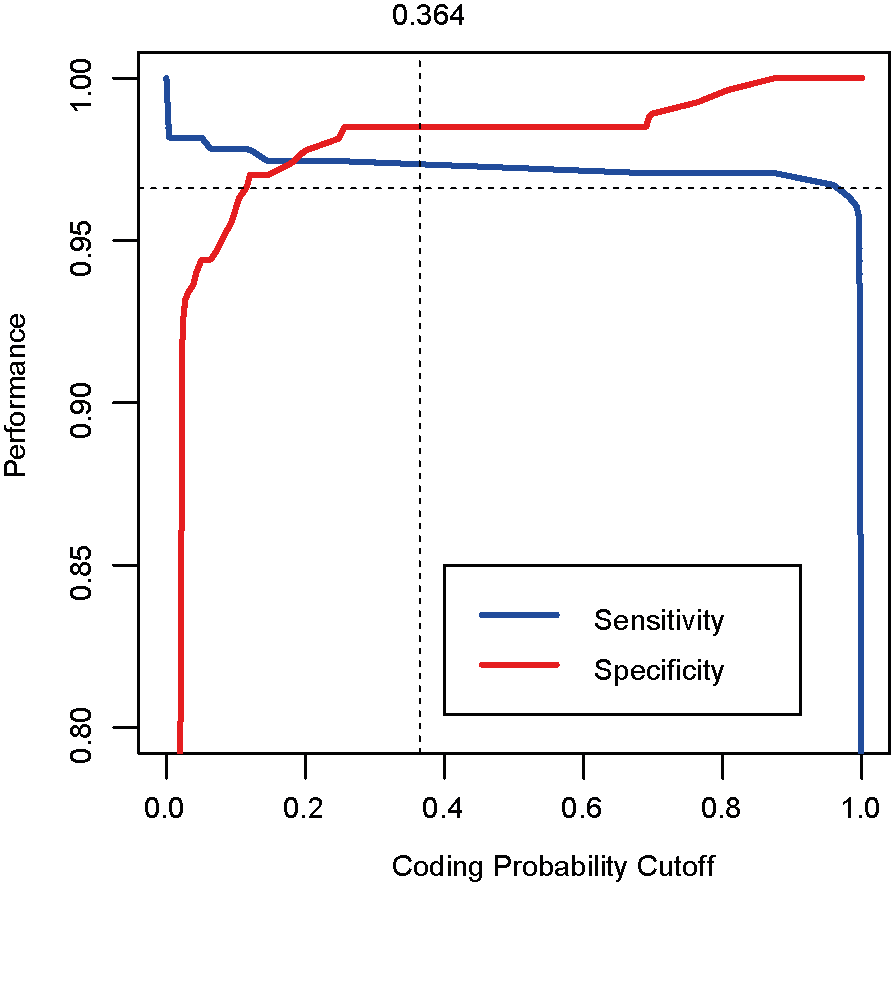


**Additional file 4: Figure S1.** Determination of optimal cutoff value to differentiate non-coding transfrags from coding ones. The optimal cutoff value was determined by R script within the CPAT package with appropriate modification to fit our circumstance.
