# Supplementary material for: Functional elucidation of the non-coding RNAs of Kluyveromyces marxianus in the exponential growth phase
Source: BMC Genomics. 2016 Feb 29;17:154. doi: 10.1186/s12864-016-2474-z (PMC4770515; doi:10.1186/s12864-016-2474-z)
Supplement: Additional file 6: Figure S2. — Average read profile of each RNA classes. Solid line indicates forward strand while dotted line indicates reverse (antisense of the forward) strand. TIS indicates transcription initiation site and TTS indicates transcription termination site. (DOC 70 kb) [file 12864_2016_2474_MOESM6_ESM.doc]

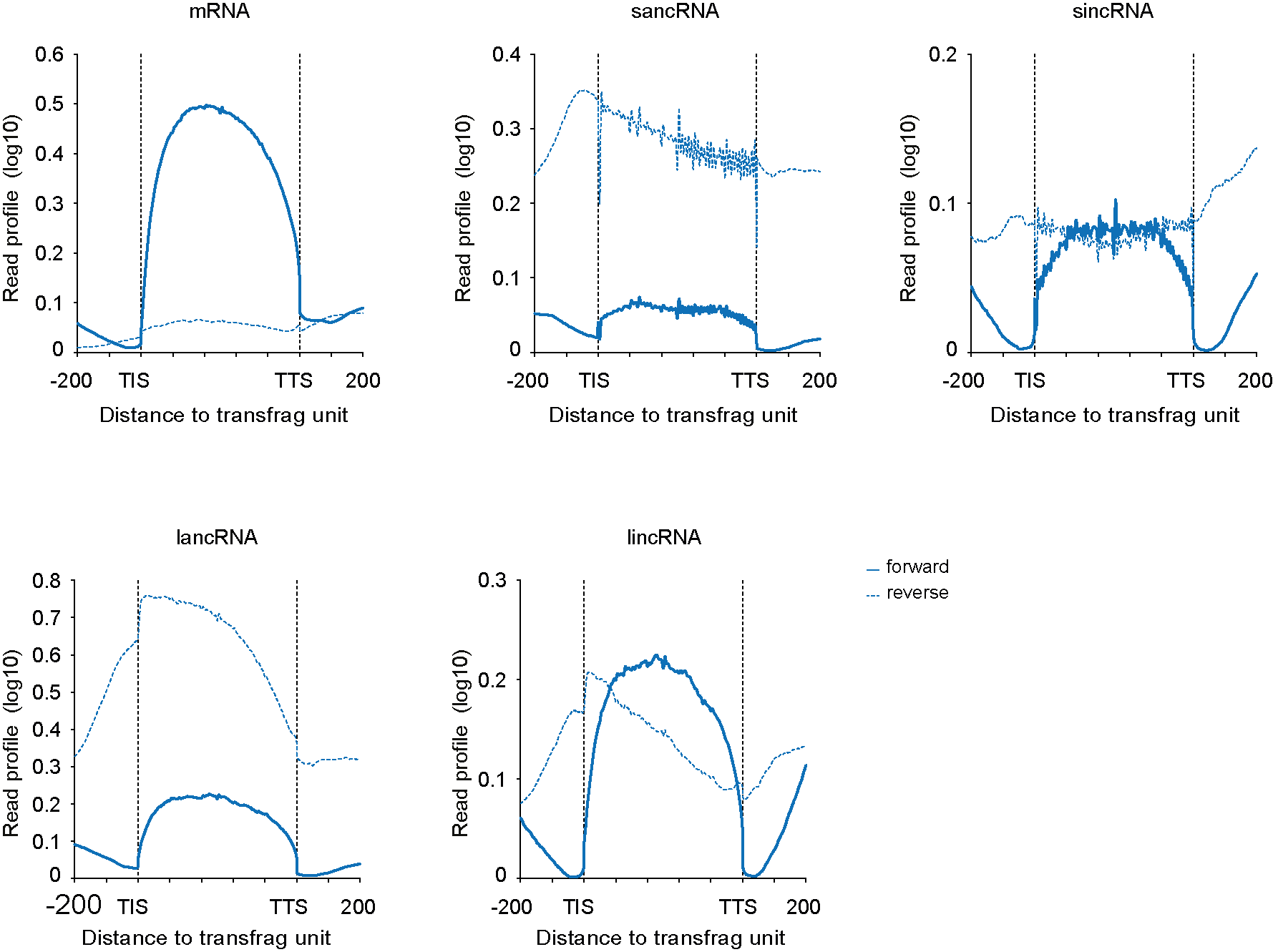


Additional file 6: Figure S2. Average read profile of each RNA classes. Solid line indicates forward strand while dotted line indicates reverse (antisense of the forward) strand. TIS indicates transcription initiation site and TTS indicates transcription termination site.
