# Supplementary material for: Functional elucidation of the non-coding RNAs of Kluyveromyces marxianus in the exponential growth phase
Source: BMC Genomics. 2016 Feb 29;17:154. doi: 10.1186/s12864-016-2474-z (PMC4770515; doi:10.1186/s12864-016-2474-z)
Supplement: Additional file 7: Figure S3. — Differential expression of transfrags. (A) Differential expression pattern of mRNA/lancRNA pairs. (B) Differential expression pattern of mRNA/sancRNA pairs. (DOC 144 kb) [file 12864_2016_2474_MOESM7_ESM.doc]

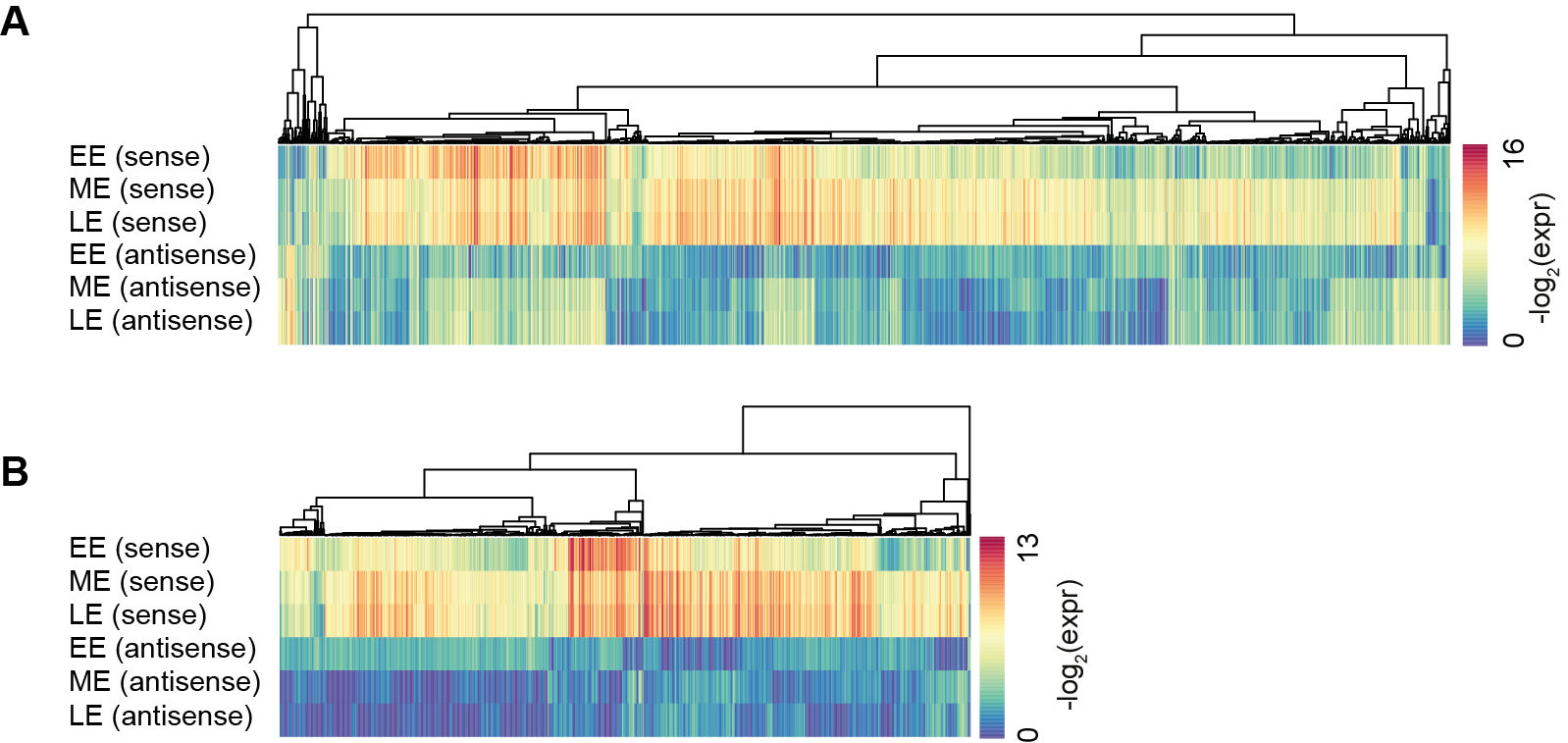


Additional file 7: Figure S3. Differential expression of transfrags. (A) Differential expression pattern of mRNA/lancRNA pairs. (B) Differential expression pattern of mRNA/sancRNA pairs.
