# Supplementary material for: Functional elucidation of the non-coding RNAs of Kluyveromyces marxianus in the exponential growth phase
Source: BMC Genomics. 2016 Feb 29;17:154. doi: 10.1186/s12864-016-2474-z (PMC4770515; doi:10.1186/s12864-016-2474-z)
Supplement: Additional file 8: Figure S4. — Significantly enriched KEGG pathways of genes with lancRNAs in S. cerevisiae. Significantly enriched KEGG pathways of genes with lancRNA which covers more than half of coding region at ME (mid exponential), ES (early stationary), and HS (heat shock) conditions. Red words indicate carbohydrate metabolism or energy metabolism pathways. In constrast to K. marxianus with no KEGG annotation, gene-pathway link information within KEGG annotation was used rather than inferred by homology search. (DOC 49 kb) [file 12864_2016_2474_MOESM8_ESM.doc]

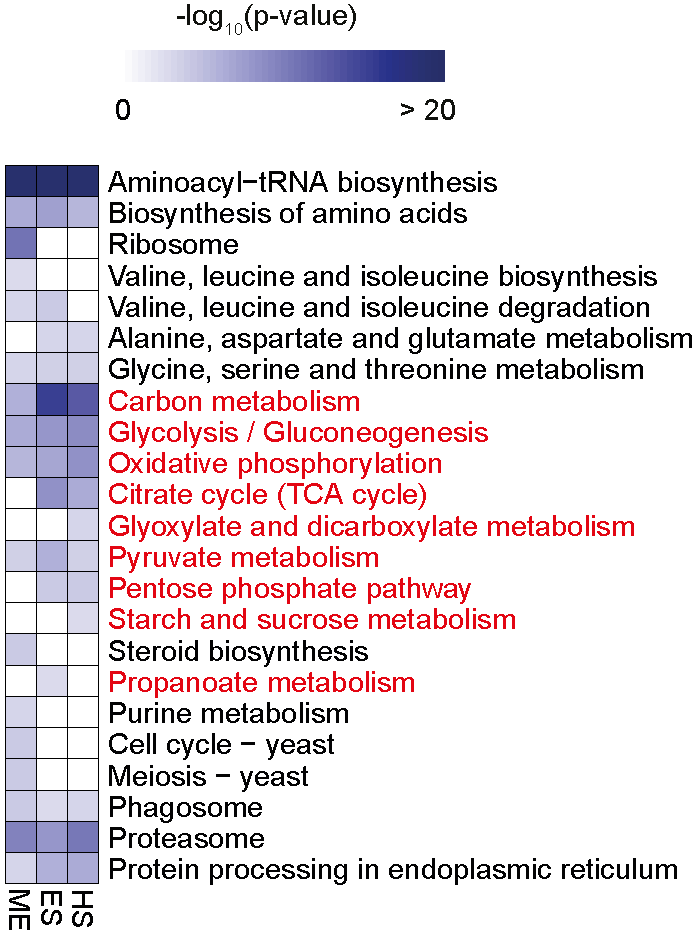


**Additional file 8: Figure S4.** Significantly enriched KEGG pathways of genes with lancRNAs in *S. cerevisiae*. Significantly enriched KEGG pathways of genes with lancRNA which covers more than half of coding region at ME (mid exponential), ES (early stationary), and HS (heat shock) conditions. Red words indicate carbohydrate metabolism or energy metabolism pathways. In constrast to *K. marxianus* with no KEGG annotation, gene-pathway link information within KEGG annotation was used rather than inferred by homology search.
